# Factors associated with inadequate urinary iodine concentration among pregnant women in Mbeya region Tanzania.

**DOI:** 10.12688/f1000research.55269.1

**Published:** 2021-08-26

**Authors:** Tedson Lukindo, Ray Masumo, Adam Hancy, Sauli E. John, Heavenlight A. Paulo, Abraham Sanga, Ramadhan Noor, Fatoumata Lankoande, Elifatio Towo, Germana H. Leyna, Gemma Bridge, Raman Bedi

**Affiliations:** 1 Tanzania Food and Nutrition Centre, 22 Barack Obama Drive, Dar es Salaam, P.O. Box 977, Tanzania; 2 Department of Epidemiology and Biostatistics, Muhimbili University of Health and Allied Sciences, Dar es Salaam, MUHAS, P.O. Box 65001, Tanzania; 3 The United Nations Children's Fund (UNICEF), The United Nations Children's Fund (UNICEF), Dar es Salaam, Tanzania, P.O. Box 4076, Tanzania; 4 Global Child Dental Fund (GCD fund), King's College London, Norfolk Building, Room G03-G03A, The Global Child Dental Fund, Surrey Street, London, WC2R 2ND, UK; 5 Barts and The London School of Medicine and Dentistry, Queen Mary University of London, Garrod Building, Turner Street, Whitechapel,, London, E1 2AD, UK; 6 King's College London, Norfolk Building, Room G03-G03A, Surrey Street, London, WC2R 2ND, UK

**Keywords:** Iodine deficiency, medium urine iodine concentration; pregnant women; socio-demographic and dietary risk factors

## Abstract

**Background: **Deficient and excess iodine intake during pregnancy can lead to serious health problems. In Tanzania, information available on iodine status during pregnancy is minimal. The aim of this study was to assess the iodine status and its association with sociodemographic factors in pregnant women in the Mbeya region, Tanzania. **Method:** A cross sectional survey involving 420 pregnant women (n=420) aged between 15-49 years registered in antenatal care clinics was conducted. Data were collected via interviews and laboratory analysis of urinary iodine concentration (UIC). **Results: **Median UIC was 279.4μg/L (+/-26.1) to 1915μg/L. Insufficient iodine intake (UIC below 150μg/L) was observed in 17.14% of participants, sufficient intake in 24.29% and 58.57% had intakes above the recommended level (>250μg/L). Rungwe district council (DC) had the highest proportion of patients (27.9%) with low iodine levels, while Chunya and Mbarali DCs had the greatest proportion of those with UIC’s, over the WHO recommended level. Fish consumption and education status were associated with increased risk of insufficient iodine while individuals in Mbalali DC aged between 35-49 years were associated with increased risk of UIC above recommended level. **Conclusion:** Both deficient and excess iodine intake remains a public health problem, especially in pregnant women in Tanzania. Therefore, educational programs on iodine intake are needed to ensure this population has an appropriate iodine intake to prevent any health risks to the mother and the unborn child.

## Introduction

Iodine deficiency is a significant global public health concern. ^1^ This element is found in hormones produced by the thyroid gland namely, triiodothyronine (T3) and thyroxine (T4), ^1, 2^ and must be consumed in the diet as it cannot be made naturally in the body. ^1^ A diet low in iodine results in deficiencies that can occur at any age. If iodine requirements are not met, the thyroid is unable to produce thyroid hormone in sufficient quantities, leading to iodine deficiency disorders (IDD) with associated functional and developmental abnormalities. ^3^

In terms of daily intake of iodine, the World Health Organization (WHO) recommends 90 μg for children aged 0-5 years; 120 μg for children aged 6-12 year; and 150 μg for those over 12 years. Pregnant and lactating women are recommended 250 μg ^4^ daily. By ensuring that individuals have an adequate intake of iodine in their diets, IDDs can be prevented. ^5^ Although cretinism is the most severe form of iodine deficiency, minor iodine deficiency can also result in reduced intellectual ability, limited work capacity due to mental and neurological impairment. ^4^ In 1994, from the 1572 million people globally who suffered from iodine deficiency (28.9% of global population), 11.2 million were affected by overt cretinism, and 43 million people had at least some degree of intellectual impairment. ^5^

In Tanzania, the most recent figures indicate that more than 40% of the population live in geographical regions prone to iodine deficiency. ^6^ The prevalence of IDD is greatest in the highland regions such as Iringa, Arusha, Mbeya, Rukwa and Ruvuma. ^7^ The Tanzania Demographic and Health Survey (TDHS) indicates that such IDD variations occur largely because of differences in the use of adequately iodised salt (15+ ppm), with households in urban areas more likely to use adequately iodised salt (81%) than those in rural areas (51%) such as the highlands. ^7^ As recommended by WHO, Iodine Global Network (IGN) and United Nations Children’s Fund (UNICEF), median Urinary Iodine Concentration (UIC) is considered the most practical biochemical marker for the assessment and monitoring of iodine nutrition in the population. ^8^ According to the National IDD survey conducted in 2004, about 25% of primary school children in Tanzania had UIC below 100 μg/L. ^6^

Universal salt iodization (USI), whereby all the salt used for human consumption is iodized, is the most used intervention to increase iodine intake. ^9^ This intervention is widespread, as 68% of households have access to iodized salt. ^10^ USI is an effective way of delivering iodine to individuals and in term, improving cognition in populations exposed to iodine deficiency. ^10, 11^ USI is also affordable, with annual costs of salt iodization estimated at USD 0.02-0.05 per child, while the costs to prevent child death is estimated at USD 1000. There are also large gains in per Disability-Adjusted Life Years (DALYs), at USD 34-36. ^12^ Before USI, it was estimated that iodine deficiency leads to losses of USD 35.7 billion in the countries affected, which is significant when compared with an estimated cost of USD 0.5 billion for USI, representing a cost benefit ratio of over 70:1. ^13^

In 1995, Tanzania legislation on USI was enacted. ^14^ This was later revised in 2010, and now, all salt consumed by animals and humans in the country is fortified with iodine. Enforcement of this legislation is challenging in Tanzania, especially in areas where small-scale salt producers operate. As a result, iodized salt is not widely available. Further, although household coverage with iodized salt is above 80% nationally, the coverage of adequately iodized salt is at 47% across Tanzania. ^15^

Nutritional deficiencies are prevalent among pregnant women in Tanzania, particularly, in areas of low socioeconomic status. However, there is a dearth of information regarding the burden of, and factors associated with iodine deficiency in this population. In 2010 and 2015, the TDHS reported that the prevalence of iodine deficiency amongst pregnant women was 54%, however, the results of the TDHS were heterogeneous across the regions. ^16, 17^ There are also concerns relating to excessive iodine consumption in pregnancy, because although high iodine intakes are well tolerated by most healthy individuals, in some, excess intake can lead to thyroid conditions such as hyperthyroidism, hypothyroidism, and/or thyroid autoimmunity. ^18^^,^^19^ As insufficient and excessive iodine consumption in pregnancy can result in negative health impacts, it is imperative to investigate current iodine levels and to assess how current USI interventions affect iodine intake, especially in highland areas of Tanzania. It is also necessary to set upper as well as lower limits for maternal iodine intake to ensure optimal health outcomes are achieved. ^19^ Given the above, this study aimed to firstly, determine the likelihood of iodine levels being above or at recommended levels in the urine (μg/L) of pregnant women in their second trimester. Secondly. to assess the likelihood of IDD or otherwise, differing across socioeconomic groups and locations in Tanzania.

## Methods

### Study design

This was a cross-sectional survey involving pregnant women aged 15-49 years registered in antenatal care clinics (ANC). Study participants were recruited in all seven district councils (DC) of the Mbeya region (Chunya district, Ileje district, Mbarali district, Mbeya urban district, Mbozi district, Momba district and the Rungwe district), from September until October 2020. The Mbeya region has 17 hospitals, 23 health centres, and 278 dispensaries, where 251 health facilities provide reproductive and child health services. The study was conducted at 42 Reproductive and Child Health (RCH) clinics in the seven districts of the Mbeya region. The selected RCH clinics provide services to approximately 1036 pregnant women.

### Study population and sample size

Pregnant women aged 15-49 years that were within their first and second trimesters (less than 28 weeks of gestation), who attended ANC in the Mbeya region were recruited into the study. Overall, 574 participants were invited to participate, from which 420 (n = 420) agreed to take part. Participants in their second trimester, a period during which fetal neurodevelopment is impacted by adequate maternal thyroid function, were included. To eliminate the effects of gestational age on thyroid hormone, participants beyond eight weeks of gestation were excluded. Additionally, individuals who did not give consent, or were not able to communicate due to illness, and those taking medication were excluded. The sample size for the whole survey was pre-calculated based on the Lwanga and Lemeshow formula. ^20^

### Sample size and sampling procedure

In the Mbeya region, 251 governmental and faith-based health facilities that provided RCH services were included in a list, which was used to randomly select health facilities per district. In the survey, 42 facilities were randomly selected to take part, with two additional sites later invited, giving a total of 44 facilities in the final survey. Probability proportional to size was performed, due to the sampling frame of public health facilities in Mbeya, to allocate the number of facilities per district in the survey. Next, pregnant women from each selected health facility were invited to take part in the research. All pregnant women attending the ANC were asked to complete an eligibility form. Those that were eligible to take part were included in a Systematic Random Sampling procedure. This was done by obtaining an accurate and complete list of the pregnant women who had attended ANC in each health facility, and randomly selecting the required number of women per facility.

### Data collection

Data were collected through interviews guided by a structured questionnaire and, laboratory analysis of urine samples. A standard structured questionnaire was constructed in English and translated into Kiswahili, a language that is spoken by almost 95% of Tanzanians (see Extended data). ^21^ To ensure the quality of the translation, back-translation was performed by independent translators and reviewed by field staff in Mbeya. Pre-testing was done to evaluate the quality of the translations in terms of comprehensibility, readability, and relevance to assess face validity.

The interviews were administered by a health professional trained in face-to-face interviews with participants, before the collection of urine samples. Initial interviews were administrated to determine various social demographic characteristics and dietary factors concerning iodine status, including participant’s age, marital status, education, household assets possessions, socioeconomic status, parity, stage of pregnancy, and dietary habits.

### Urine sample collection and laboratory analysis

Most of iodine consumed in the diet (90%) is lost through urine. As such UI is used as an indicator of iodine intake, expressed either as (μg/L), in terms of its relationship to creatinine excretion (μg iodine/g creatinine), or as 24-hour excretion (μg/day), termed urinary iodine excretion (UIE). It is impractical to collect 24-hour samples in field studies, therefore UI acts as a practical alternative to assess UI (expressed as the median, in μg/L) in a representative sample of the target group. After interviews were completed, a trained member of nursing staff took spot urine samples from consented participants. Urine samples were collected in a disposable plastic screw caped 100ml urine container. The urine samples were transported to a temporary laboratory for processing and shipment to central Tanzania Food and Nutrition Centre (TFNC) laboratory for analysis. At the temporary laboratory, the urine samples were transferred into screw-capped plastic vials and frozen at −20°C until shipped to TFNC laboratory for analysis. The urine samples were analyzed using the ammonium persulfate digestion method, as previously described by Sandell-Kolthoff reaction. ^22^ TFNC laboratory is registered and successfully participated in the quality assurance program for Ensuring the Quality of Urinary Iodine Procedures (EQUIP) ^23^ offered by the Centres for Disease Control and Prevention (CDC), Atlanta, Georgia, USA. The assay accuracy was assessed using reference quality-control urine specimens obtained from the CDC. The assay detection limit was <5.0 μg/L with the coefficient of variation <10%, when compared to the reference method. ^23^

### Variables

Outcome/response variable

Median UCI as a response variable was split into three categories as per WHO recommended level of iodine micronutrient. A new variable called medium urine iodine concentration (MUIC) was developed to indicate the level of iodine in urine (μg/L) (see underlying data). ^21^

MUIC 1 (Iodine <150μg/L) = Insufficient/deficiency iodine

MUIC 2 (150< Iodine <249 μg/L) = Sufficient/adequacy iodine

MUIC 3 (Iodine >250 μg/L) = Excessive iodine intake

Independent variables/predictors

The study includes a set of independent variables to understand the extent and variations between the levels of iodine micronutrients among the participants. Socio-demographic variables assessed included age, residency (district), education level, occupation status, number of pregnancies, visits to the ANC and, upper mid-arm circumference (MUAC), which is the most accurate way to measure fat-free mass outside of a laboratory. Household wealth was also assessed. To do so, durable household assets that indicate wealth such as a radio, television, and telephone were recorded as (1) “available and in working condition” or (0) “not available and/or not in working condition.” Principal component analysis, PCA was then conducted to categorize households into five quartiles of wealth, with 1 being the lowest and 3 the highest. Diet, in specific consumption of certain foods, such as fish, dairy products, processed meat and, refined and baked foods were also assessed among the participants, using 24-hour recalls.

### Data analysis

The data were analyzed using Stata v 15.1(RRID: SCR_012763). Stata is a proprietary software but an open-access alternative in which the sequence could have been generated is Microsoft Excel (RRID: SCR_016137). Descriptive statistics were used to summarize the data of study participants. Pearson’s chi-square test and *p*-values were used to test for the significance of each of the potential risk factors in bivariate analysis. Multinomial logistic regression models were used to adjust for cofounders and predict the true association between the dependent and independent variables. All tests were two-tailed, and the significance level was set at p ≤ 0.05.

### Ethical approval and informed consent

Ethical clearance was obtained from the National Institute for Medical Research (NIMR) with reference number NIMR/HQ/R.8a/Vol. IX/2589 and appropriate authorization was given from the Regional, Council and health facility level. All eligible subjects were given information about the survey and were asked to sign a written informed consent form before participation.

## Results

### Descriptive of the study participants

In this study, 420 agreed to participate (response rate of 73 %), with the mean age of 25.49 (± 6.37) years. In terms of demographics, 70% of participants had primary education, 75% has been pregnant more than once, 68% reported that they consumed fish and, more than 90% consumed dairy products. Improved source of water was reported by 71% of the participants ( Table 1).

**Table 1.** Frequency distribution of the study participant in Mbeya (n = 420).

Variables	Category	% (n)
Age group	15-24	52.18 (215)
	25-34	35.68 (147)
	35-49	12.14 (50)
Education level	No formal education	8.10 (34)
	Primary education	71.67 (301)
	Secondary and above	20.24 (85)
Wealth Index	1quantile	33.3 (140)
	2quantile	33.3 (140)
	3quantile	33.3 (140)
Marital status	Married	56.67 (238)
	Cohabit	31.67 (133)
	Single	9.29 (39)
	Divorced	2.38 (10)
Occupational status	Formal employment	3.57 (15)
	Self-employment	84.52 (355)
	Not employed	11.90 (50)
Antenatal care center (ANC) visit	1 visit	38.81 (163)
	2-3 visits	53.81 (226)
	More than 3 visits	7.38 (31)
Residence	Chunya District Council	10.71 (45)
	Mbeya District Council	23.10 (97)
	Mbarali District Council	22.14 (93)
	Kyela District Council	11.90 (50)
	Rungwe District Council	16.19 (68)
	Busokelo District Council	7.86 (33)
	Mbeya City	8.10 (34)
Number of pregnancies	Primiglavida	24.76 (104)
	Multiglavida	75.24 (316)
Type of water source	Improved	71.90 (302)
	Unimproved	28.10 (118)
Mid-upper arm circumference (MUAC) categorization	MUAC < 23cm	3.81 (16)
	MUAC ≥ 23cm-MUAC < 33cm	90.19 (383)
	MUAC ≥ 33cm	5.0 (21)
Consumption of fish	No	68.3 (287)
	Yes	31.7 (133)
Consumption of Dairy products	No	90.7 (381)
	Yes	9.8 (39)
Consumption of Processed meat	No	97.6 (410)
	Yes	2.4 (10)
Urinary Iodine Concentration (UIC) categorization	Insufficient (UIC 0–149 μg/l)	17.14 (72)
	Sufficient (UIC 150–249 μg/l)	24.29 (102)
	Above recommended (>250μg/l)	58.57 (246)

### Urinary iodine concentration (UIC)

The median UIC in the present study was 279.4μg/L, and it ranged from 26.1-1915μg/L. According to the UIC results, 17.14% of participants had an insufficient iodine intake, 24.29% had sufficient/adequate urine iodine concentration, and 58.57% had above the recommended level of iodine in urine ( Table 1).

### Bivariate analysis

Of 215 participants aged between 15-24 years, 17% had UIC (0–149 μg/l) that would be considered inadequate, and 55.8% had UIC (>250 μg/l) above the recommended levels. Table 2 presents a cross-tabulation of the prevalence of median UIC, MUIC and various independent factors. Chunya and Mbarali DCs have the highest percentage (above 70%) of the WHO recommended UIC among the participants in the sample. Rungwe DC had the highest percentage (27.9%) of participants with inadequate urine iodine concentrations. From the 133 participants who had fish in their diet, UIC was inadequate in 23%, adequate in 19.4%, and 56.9% had above the recommended level.

**Table 2.** Predictors of urine iodine concentration level (MUIC) among pregnant women in Mbeya (n = 420).

		Insufficient (Urinary Iodine Concentration (UIC) 0–149 μg/l)	sufficient (UIC 150–249 μg/l)	Above recommended (>250μg/l)	Chi-square (X ^2^)	P value
Variable	Category	% (n)	% (n)	% (n)		
Age group	15-24	17.21(37)	26.98 (58)	55.81(120)	4.0208	0.403
	25-34	16.33 (24)	22.45(33)	61.22(90)		
	35-49	14.00(7)	16.00 (8)	70.00 (35)		
Education level	No formal education	26.47 (9)	17.65 (6)	55.88 (19)	4.314	0.634
	Primary education	15.61 (47)	24.92 (75)	59.47 (179)		
	Secondary and above	16	21	48		
Wealth Index	1quantile	20.71 (29)	26.43 (37)	52.86 (74)	4.7325	0.316
	2quantile	15.71 (22)	20.00 (28)	64.29 (90)		
	3quantile	15.00 (21)	26.43 (37)	58.57 (82)		
Marital status	Married	18.49 (44)	22.27 (53)	59.24 (141)	2.71	0.838
	Cohabit	15.04 (20)	28.57 (38)	56.39(75)		
	Single	17.95 (7)	23.08 (9)	58.97 (23)		
	Divorced	10.0 (1)	20.0 (2)	70.0 (7)		
Occupational status	Formal employment	20.0(3)	33.33(5)	46.67 (7)	4.132	0.388
	Self-employment	17.46 (62)	22.54 (80)	60.0(213)		
	Not employed	14.0 (7)	34.0 (17)	52.0 (26)		
Antenatal care center (ANC) visit	1 visit	17.18(28)	23.93 (39)	58.90 (96)	3.3699	0.498
	2-3 visits	18.58 (42)	24.78 (56)	56.64 (128)		
	More than 3 visits	6.45 (2)	22.58 (7)	70.97 (22)		
Residence	Chunya DC	6.67 (3)	22.22(10)	71.11 (32)	31.987	0.001
	Mbeya DC	21.62 (21)	32.99 (32)	45.36 (44)		
	Mbarali DC	11.83 (11)	13.98 (13)	74.19(69)		
	Kyela DC	12.0(6)	20.0 (10)	68.0 (34)		
	Rungwe DC	27.94 (19)	25.0 (19)	47.06 (32)		
	Busokelo DC	24.24 (8)	27.27 (9)	48.48 (16)		
	Mbeya city	11.76 (4)	32.35 (11)	55.8 (19)		
Number of pregnancies	Primiglavida	16.35 (17)	22.12(23)	61.54 (64)	0.527	0.768
	Multiglavida	17.71 (55)	25.0 (79)	57.59 (182)		
Type of water source	Improved	18.54 (56)	23.51 (71)	57.95 (175)	0.567	0.457
	Unemployed	13.56 (16)	26.27 (31)	60.17 (71)		
Mean- upper arm circumference (MUAC) categorization	MUAC < 23cm	12.50 (2)	18.75 (3)	68.75(11)	0,987	0.912
	MUAC ≥ 23cm-MUAC < 33cm	17.49(67)	24.28 (91)	58.22 (223)		
	MUAC ≥ 33cm	14,29(3)	28.57 (6)	57.14 (12)		
Consumption of fish	No	13.77 (38)	26.81 (74)	59.42 (164)	7.5619	0.023*
	Yes	23.61(34)	19.44 (28)	56.94 (82)		
Consumption of Dairy products	No	17.19(60)	23.78 (83)	59.03 (206)	0.2912	0.865
	Yes	16.90(12)	26.76 (16)	56.34 (40)		
Consumption of Processed meat	No	17.27 (71)	23.84 (98)	58.88 (242)	2.0475	0.359
	Yes	11.11 (1)	44.44 (4)	44.44(4)		
Consumption of refined and baked	No	18.92(14)	24.32 (18)	56.76 (42)	0.2158	0.898
	Yes	16.76(58)	24.28(84)	58.96 (204)		

### Multivariate analysis

The fitted models and the estimated effects from the multivariate analysis are presented in Table 3. The chi-square model (63.51) was 0.0176, with p < 0.05.

**Table 3.** Multinomial logistic regression models for iodine intake among pregnant women in Mbeya (n = 420).

Model fitting information			
Model	-2logLikelihood	Chi-Square	P-value
Intercept Only	391.00997		
Final	359.25317	63.51	0.0176

Parameters estimates						
Dependent	Independent			95% confidence interval for Exp(B)		
	Variable	Category	Exp(B)	Lower bound	Upper bound	P- value
**Insufficient**	Consumption of fish	No				
		Yes	2.60	1.31	5.15	0.006 *
	Consumption of Dairy products	No				
		Yes	0.96	0.41	2.28	0.940
	Consumption of Processed meat	No				
		Yes	0.32	0.03	3.19	0.334
	Consumption of refined and baked	No				
		Yes	0.79	0.33	1.91	0.609
	Residence	Mbeya district council (DC)	1	1	1	
		Chunya DC	0.38	0.08	1.65	0.199
		Mbarali DC	1.15	0.38	3.44	0.793
		Kyela DC	0.85	0.25	2.92	0.809
		Rungwe DC	2.43	0.95	6.19	0.061
		Busokelo DC	1.79	0.52	6.11	0.351
		Mbeya city	0.77	0.18	3.24	0.732
	Age group	15-24				
		25-34	1.11	0.50	2.44	0.782
		35-49	1.45	0.42	4.98	0.553
	Wealth Index	1quantile				
		2quantile	1.62	0.62	4.26	0.321
		3quantile	1.28	0.42	3.90	0.663
	Education level	No formal education				
		Primary education	0.29	0.08	0.99	0.049 *
		Secondary and above				
	Mean upper arm circumference (MUAC) categorization	MUAC < 23cm				
		MUAC ≥ 23cm-MUAC < 33cm	1.27	0.18	8.90	0.807
		MUAC ≥ 33cm	1.17	0.10	13.31	0.896
	Number of pregnancies	Primiglavida				
		Multiglavida	0.83	0.34	2.01	0.683
**Above recommended**	Consumption of fish	No				
		Yes	1.24	0.71	2.15	0.438
	Consumption of Dairy products	No				
		Yes	0.90	0.46	1.73	0.754
	Consumption of Processed meat	No				
		Yes	0.50	0.11	2.31	0.379
	Consumption of refined and baked	No				
		Yes	0.99	0.50	1.96	0.998
	Residence	Mbeya DC				
		Chunya DC	2.05	0.84	4.96	0.110
		Mbarali DC	4.09	1.85	9.01	0.000 *
		Kyela DC	2.15	0.88	5.23	0.089
		Rungwe DC	1.49	0.67	3.29	0.321
		Busokelo DC	1.55	0.56	4.26	0.390
		Mbeya city	1.45	0.54	3.94	0.456
	Age group	15-24				
		25-34	1.47	0.81	2.69	0.201
		35-49	2.51	0.99	6.33	0.050 *
	Wealth Index	1quantile				
		2quantile	1.41	0.66	3.03	0.367
		3quantile	2.08	0.91	4.71	0.079
	Education level	No formal education				
		Primary education	1.04	0.37	2.94	0.929
		Secondary and above	1.18	0.35	3.95	0.777
	MUAC categorization	MUAC < 23cm				
		MUAC ≥ 23cm-MUAC < 33cm	1.01	0.25	4.05	0.985
		MUAC ≥ 33cm	0.74	0.13	4.27	0.745
	Number of pregnancies	Primiglavida				
		Multiglavida	0.64	0.32	1.26	0.203

The reference category contains: sufficient/adequate/sufficient iodine concentration.

* Represents p value: *p<0.05.

The predicted probability of having inadequate urine iodine level was 2.60 (95% CI 1.31-5.15), in participants who consumed freshwater fish. Additionally, inadequate iodine level was 0.29 (95 % CI 0.08-0.99) in participants who had a primary school education. The probability of having increased iodine levels amongst participants living in Mbarali DC was 4.09 (95% CI 1.85- 9.01), whilst for individuals aged 35-49 years, the probability was 2.51 (95% CI 0.99-6.33). The likelihood of having an inadequate iodine level based on DC, resulted in a significant likelihood of having above recommended iodine level for individuals in Mbarali DC [4.09 (95% CI 1.85-9.01)], whilst those living in Rungwe DC had a significant (borderline) likelihood of having inadequate iodine levels [2.43 (95% CI 0.95-6.19)].

## Discussion

This is the first population-based cross-sectional study to assess the magnitude of iodine status and the association with socio-demographic factors and diet in Tanzanian pregnant women. The findings of the study are important since iodine deficiency is the most prevalent micronutrient deficiency, affecting 28.9% of the world population, ^24^ particularly affecting women living in developing countries. ^25^ Iodine deficiency in Tanzania is also high with the most recent figures indicating that more than 40% of the population in the country lives in geographical regions prone to iodine deficiency. ^6^ However, this data is largely outdated, as more recent data as well as the most recent efforts to reduce iodine deficiency have focused on primary school children in Tanzania. ^6^ Whilst the iodine micronutrient status among pregnant women has been overlooked in recent years.

In this study, pregnant women living in the Mbarali district were more likely to be above the recommended level of UIC [4.09 (95% CI 1.85-9.01)], particularly among individuals aged between 35-49 years [2.51 (95% CI 0.99-6.33)]. Thus, it is important to monitor thyroid function and its associated disorders in this population. Contrary to these results, in 2010, a reanalysis of the Tanzania demographic and health survey reported 54% of pregnant women with iodine deficiency. ^16^ The discrepancy could be attributed to differences in study methodologies as well as the 10-year lapse between the studies, since significant USI interventions were applied in this period.

In this study a strong association between consumption of freshwater fish and UIC of <150μg/l [odd ratio = 2.60 (95% CI 1.31-5.15)], indicated that pregnant women who consumed fish were at higher risk of iodine deficiency. This finding could be explained by the notion that iodine levels in freshwater fish depend on the locality and the regularity of consumption of fish. ^26, 27^ Moreover, during pregnancy there are variations in the functionality of the thyroid. This can increase the risk of inadequate iodine intake for some mothers. As such, predicting UIC based on usage of iodized salt alone, may not be accurate. ^28– 30^ Other studies have documented that freshwater fish may contain Iodine in levels that can improve daily Iodine intake. ^26^

In countries with successful USI programs, studies have reported an optimal median UIC in pregnant women. As such, USI remains the most cost-effective strategy for achieving reduced IDD. ^31, 32^ However, the full implementation of USI remains a challenge in many sub–Saharan African countries including Tanzania, ^33^ largely due to the lack of adequate enforcement and, the inadequate monitoring of small-scale salt producers who often do not comply with USI legislation. ^6^

This analysis also indicated that pregnant women who had a primary school education were at higher risk of iodine deficiency [odd ratio= 0.29 (95% CI 0.08-0.99)], However, further studies are needed to investigate this association. Excessive iodine intake in pregnant women is also an important area of current research. ^34, 35^ The WHO recommended an increased iodine intake for pregnant women, although evidence is weak. ^36^ However, detrimental effects from more than adequate and excessive iodine intake have been reported in general populations. ^37– 39^ Shi et al. have reported on the associations between UIC and thyroid health among pregnant women and recommend a lower limit for maternal iodine intake during pregnancy than that currently advised by the WHO. ^40^ This is also an area in need of further investigation. The question remains if pregnant women in Mbarali district should continue using iodinated salt, and if so at what concentration.

The strength of this study is in its large population-based sample size. The study limitations are as follows, first, the use of UIC to determine individual iodine status could be limited due to the potential for misclassification of participants because of day-to-day variations. Second, UIC reflects recent iodine intake or exposure rather than chronic individual iodine status. Additionally, the use of iodized salt was not assessed in this study. Finally, it would have been useful to have a non-pregnant control group to help ascertain whether lower UI concentrations during pregnancy could be attributed to pregnancy itself or the diet.

## Conclusion

The aim of this study was to explore iodine levels in pregnant women living in the Mbeya region of Tanzania. Findings indicate that 17.14% of participants had an insufficient iodine intake whilst, 24.29% had sufficient/adequate urine iodine concentration, and 58.57% had above the recommended levels of iodine. There were differences found between district councils, with the Rungwe DC having the highest percentage (27.9%) of participants with inadequate urine iodine concentrations. Protective factors for IDD included consumption of freshwater fish and having a primary education, whilst factors increasing the risk of excessive iodine intake included being older in age (35-49 years). Due to these findings, this study recommends strategic efforts to ensure that the current USI program addresses the problem of iodine deficiency in pregnant women, and monitor excessive iodine exposure that might have detrimental effects during pregnancy.

## Data availability

### Underlying data

Open Science Framework (OSF): Factors associated with inadequate urinary iodine concentration among pregnant women in Mbeya region Tanzania.

DOI: https://osf.io/7ysb9/. ^21^

This project contains the following underlying data:

• MBMNS_MUIC10082021: This is the SPSS database file that contained all the laboratory assessment variables for the medium urine iodine concentrations.

This project also contains the following extended data:

• Questionnaire English version: This file contains all the questions used to interview pregnant women in Mbeya.
• Questionnaire Swahili version: This file is the Swahili version of Questionnaire.

Data are available under the terms of the Creative Commons Zero "No rights reserved" data waiver ( CC0 1.0 Public domain dedication).

## Author contributions

Conceptualization, TL, RM., AH. SEJ and GHL; project administration and resources, AS, RN, FK and GHL; formal analysis and writing—original draft, TL, RM, AH, SEJ, HAP, AS, RN, FK, ET and GHL; reviewed and edited the manuscript. GB and, RB. All authors: Reviewed and agreed upon the final manuscript.
