# Factors associated with inadequate urinary iodine concentration among pregnant women in Mbeya region Tanzania.

**DOI:** 10.12688/f1000research.55269.5

**Published:** 2024-08-22

**Authors:** Tedson Lukindo, Ray Masumo, Adam Hancy, Sauli E. John, Heavenlight A. Paulo, Abraham Sanga, Ramadhan Noor, Fatoumata Lankoande, Elifatio Towo, Germana H. Leyna, Gemma Bridge, Raman Bedi

**Affiliations:** 1 Tanzania Food and Nutrition Centre, 22 Barack Obama Drive, Dar es Salaam, P.O. Box 977, Tanzania; 2 Department of Epidemiology and Biostatistics, Muhimbili University of Health and Allied Sciences, Dar es Salaam, MUHAS, P.O. Box 65001, Tanzania; 3 The United Nations Children's Fund (UNICEF), The United Nations Children's Fund (UNICEF), Dar es Salaam, Tanzania, P.O. Box 4076, Tanzania; 4 Barts and The London School of Medicine and Dentistry, Queen Mary University of London, Garrod Building, Turner Street, Whitechapel,, London, E1 2AD, UK; 5 Global Child Dental Fund (GCD fund), King's College London, Norfolk Building, Room G03-G03A, The Global Child Dental Fund, Surrey Street, London, WC2R 2ND, UK; 6 King's College London, Norfolk Building, Room G03-G03A, Surrey Street, London, WC2R 2ND, UK

**Keywords:** Iodine deficiency, medium urine iodine concentration; pregnant women; socio-demographic and dietary risk factors

## Abstract

**Background:**

Insufficient and above WHO-recommended levels of iodine intake during pregnancy can lead to serious health outcomes. This study aimed to assess median urine iodine concentration and its associated risk factors among pregnant women in the Mbeya region, Tanzania.

**Method:**

A cross sectional survey involving 420 pregnant women (n=420) aged 15-49, registered in Reproductive and Child Health Clinics was conducted. Socio-demographic and dietary factors were assessed by structured questionnaire and the urine samples were analyzed using the ammonium persulfate digestion method.

**Results:**

Median urinary iodine concentration (mUIC) was 279.4μg/L and it ranged from 26.1 to 1915μg/L. Insufficient mUIC (below 150μg/L) was observed in 17.14% of participants, sufficient mUIC was 24.29% and 58.57% had mUIC above the recommended level (>250μg/L). Sample women who reported consuming fish in the last 24 hours had an increased risk of insufficient mUIC [Adjusted OR= 2.60 (95%CI 1.31-5.15)] while the risk was lower for those who attended at least primary education [AOR= 0.29 (CI 0.08-0.99)]. Further, sample women resident in Mbarali district, in the oldest age group (35-49) and having a higher socio-economic status were associated with an increased risk of having MUIC above recommended level [AOR=4.09 (CI 1.85-9.010], [AOR=2.51 (CI 0.99-6.330] and, [AOR=2.08 (CI 0.91-4.71) respectively.

**Conclusion:**

This study demonstrated a significant association between geographical, age and socio-economic factors and median urine iodine concentration above the WHO-recommended level. Further, this study found association between inadequate iodine in diet and insufficient median urine iodine concentration. Therefore, educational programs on iodine intake should be strengthened.

## Introduction

Iodine insufficiency is a significant global public health concern. ^1^ This element is constituent of hormones produced by the thyroid gland namely, triiodothyronine (T3) and thyroxine (T4), ^1^^,^^2^ and must be consumed in the diet as it cannot be made naturally in the body. ^1^ A diet low in iodine results in insufficiency that can occur at any age. If iodine requirements are not met, the thyroid is unable to produce thyroid hormone in sufficient quantities, leading to iodine deficiency disorders (IDD) with associated dysfunctional and developmental abnormalities. ^3^

The daily iodine intake recommended by the World Health Organization (WHO) is 90 μg for children aged 0-5 years; 120 μg for children aged 6-12 year; and 150 μg for those over 12 years. Pregnant and lactating women the recommended iodine intake is 250 μg daily. ^4^ By ensuring that individuals have an adequate intake of iodine in their diets, IDDs can be prevented. ^5^ Although cretinism is the most severe form of iodine insufficiency, minor iodine deficiency can also result in reduced intellectual ability, limited work capacity due to mental and neurological impairment. ^4^ In 1994, about 1.6 billion people globally (28.9% of global populations) were suffering from iodine deficiency, 11.2 million were affected by overt cretinism, and 43 million people had at least some degree of intellectual impairment. ^5^

In Tanzania, the most recent figures indicate that more than 40% of the populations live in geographical regions prone to iodine deficiency. ^6^ Previous studies have reported that the southern highlands of Tanzania were areas with a high prevalence of endemic goiter. ^7^^,^^8^ In 1986, Wachter and colleagues investigated the prevalence of goiter before iodine supplementation program in the Southern highlands of Tanzania among 560 schoolchildren aged between 6 to 19 years old and found that 512 children had goitre (a prevalence of 90%). Goitre decreased to 60% by 1995 and further to 17% in 2004. ^6^ In the 1990s there was a worldwide progress in reducing IDD through universal salt iodization (USI) legislation passed in Tanzania and many other countries. In 2000, more than 28 counties reduced goiter by more than 20% through USI. ^9^ The Tanzania Demographic and Health Survey (TDHS) indicates that such IDD variations occur largely because of differences in the use of adequately iodised salt (15+ ppm), with households in urban areas more likely to use adequately iodised salt (81%) than those in rural areas (51%) such as the highlands. ^9^ As recommended by WHO, Iodine Global Network (IGN) and United Nations Children’s Fund (UNICEF), median Urinary Iodine Concentration (mUIC) is considered the most practical biomarker for the assessment and monitoring of iodine nutrition status in the population. ^10^ According to the National IDD survey conducted in 2004, about 25% of primary school children in Tanzania had UIC below 100 μg/L. ^6^

For USI, where all the salt meant for human consumption is iodized, is the most practical intervention strategy to increase iodine intake. ^11^ Evidence from multiple sources indicates widespread of USI, as 68% of households have access to iodized salt. ^12^ USI is an effective way of delivering iodine to individuals and in term, improving cognition in populations exposed to iodine deficiency. ^12^^,^^13^ USI is also affordable, with annual costs of salt iodization estimated at USD 0.02-0.05 per child, while the costs to prevent child death are estimated at USD 1000. There are also large gains in per Disability-Adjusted Life Years (DALYs), at USD 34-36. ^14^ Before USI, it was estimated that iodine deficiency leads to losses of USD 35.7 billion in the countries affected, which is significant when compared with an estimated cost of USD 0.5 billion for USI, representing a cost benefit ratio of over 70:1. ^15^

Tanzanian legislation on USI was enacted in 1995. ^16^ This was later revised in 2010, and now, all salt consumed by animals and humans in the country is fortified with iodine. Enforcement of this legislation is challenging in Tanzania, especially in areas where small-scale salt producers operate. As a result, iodized salt is not widely available. Further, although household coverage with iodized salt is above 80% nationally, the coverage of adequately iodized salt is at 47% across Tanzania.

Although USI is arguably the successful health intervention in global history, legal regulations on salt production are rarely sufficient to guarantee dietary change among rural populations that consume mainly subsistence food products. ^17^ In 2005 UNICEF documented that in many African countries less than thirty per cent of households consume iodized salt despite universal legislation, and even iodized salt may be insufficient to reduce IDD in populations whose diets contain substantial amounts of iodine-depleting foods. ^18^ With the respect to the estimated more than 10% of Tanzania households which remain at risk in spite of salt iodization legislation, the magnitudes of micronutrients deficiencies among pregnant women in Tanzania, particularly, in rural areas and low socioeconomic status justify more research. There is a dearth of information regarding the burden of, and factors associated with iodine deficiency among pregnant women. In 2010 and 2015, the TDHS reported that the prevalence of iodine deficiency amongst pregnant women was 54%, however, the results of the TDHS were heterogeneous across the regions. ^19^^,^^20^ There are also concerns relating to excessive iodine intake in pregnancy, because although high iodine intakes are well tolerated by most healthy individuals, in some, excess intake can lead to thyroid conditions such as hyperthyroidism, hypothyroidism, and/or thyroid autoimmunity. ^21^^,^^22^ As insufficient and excessive iodine consumption in pregnancy can result in negative health impacts, it is imperative to investigate current iodine levels and to assess how current USI interventions affect iodine intake, especially in highland areas of Tanzania. The findings of the present study could be useful especially at regional or local salt facility levels for the future decision making on whether levels of iodine added to fortified salt should be increased or decreased, ^22^ Given the above, this study aimed to firstly, determine the likelihood of iodine levels being above or at recommended levels in the urine (μg/L) of pregnant women in their second trimester. Secondly, assess the likelihood of IDD or otherwise, differing across socioeconomic groups and locations in Tanzania.

## Methods

### Study site

**Figure 1.**
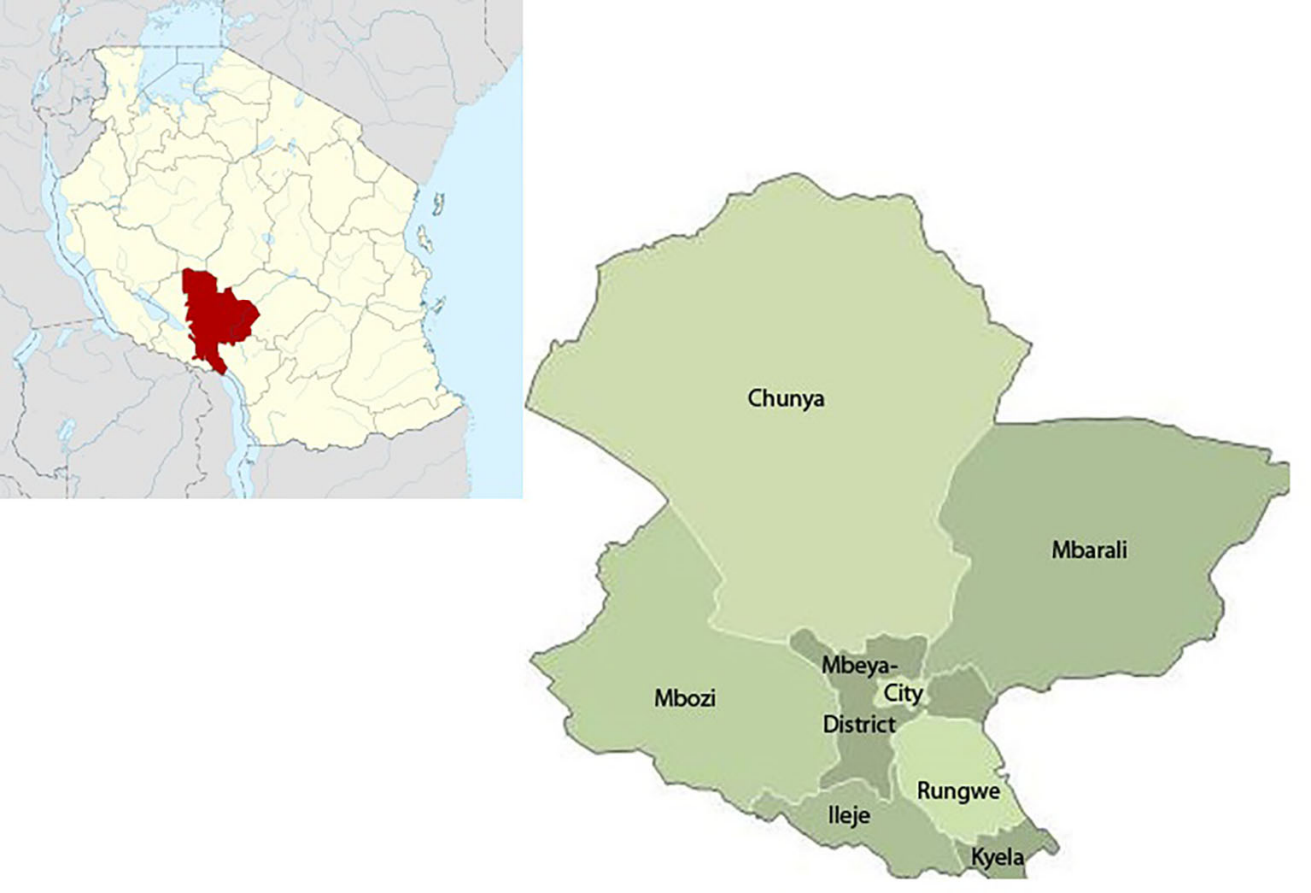
Map of Tanzania and Mbeya region.

Mbeya Region is located in the south-western corner of the southern highlands of Tanzania ( Figure 1). The Region lies between latitude 70° and 90° 31’ south of the Equator and between longitude 32° and 35° east of Greenwich. The economy of Mbeya is based mainly on agriculture. Agriculture contributes most of the Region’s cash income mainly from maize, sorghum, cassava, beans and pigeon peas’ production. Generally, annual rainfall varies from 650 mm in Usangu plains and Chunya to 2600 mm on the Northern shores of Lake Nyasa and in the highlands. The Mbeya region has a population of 2,707,410 and, in 2020 the region had 318 health facilities of which 17 were hospitals, 23 health centers, and 278 dispensaries, with 251 of the health facilities (both government, private and faith-based organizations) providing reproductive and child health services.

### Study design

A cross-sectional survey of 420 pregnant women (gestation age below 28 weeks) registered at the Reproductive and Child Health Clinics (RCH) from the seven districts of the Mbeya Region. This manuscript is part of the project on improving maternal and adolescent nutrition (IMAN) in Mbeya supported by the UNICEF-Tanzania and the Ministry of Health- Tanzania. The study was carried out from September 2020 to October 2020. This study was conducted in 42 RCH across the seven districts of Mbeya region. The allocation of RCH per district for was based to probability proportional to size: Mbeya District Council (n = 11); Chunya District Council (n = 4); Mbeya City (n = 3); Mbarali District Council (n = 8); Kyela District Council (n = 6); Rungwe District Council (n = 7) and, Busekelo District Council (n = 4). The selected RCH clinics were estimated to provide services to approximately 1036 pregnant women.

### Study population

All pregnant women aged 15-49 years who attended the selected RCH clinics within their first and second trimesters (less than 28 weeks of gestation) were invited to participate in the study. A total of 574 pregnant women were eligible, and 420 agreed to take part. Pregnant women who refused to consent and those who were unable to communicate due to illness or taking medication were excluded from the study. Participants in their second trimester, a period during which fetal neurodevelopment is impacted by adequate maternal thyroid function, were included. To eliminate the effects of gestational age on thyroid hormone, participants beyond eight weeks of gestation were excluded.

### Sample size and sampling procedure

The prevalence of iodine deficiency among women of reproductive age reported in Tanzania was estimated at 40%. Based on this figure and the population of pregnant women in this region, a sample size of 574 was calculated using the Lwanga and Lemeshow formula ^23^ with: margin error of 5%; confidence level of 95%; design effect of 1.5 and; an additional of 10% to account for non-response. Only 420 pregnant women agreed to participate and this sample size was considered satisfactory assuming intracluster correlation coefficient (ICC) of 0.10 and, a power of 80%.

The sampling procedure involved two steps: First, a list of 251 government, private and faith-based health facilities providing RCH services in Mbeya region was obtained and used in a random selection of the health facilities to be involved in the study from each district. Given the sampling frame of health facilities in Mbeya, probability proportional to size was performed to allocate the number of facilities per District for inclusion in the survey. Out of 251 health facilities that offer RCH services (eligibility criteria) in Mbeya *,* forty two facilities were randomly selected for the study. An additional two reserved clusters were included in the survey. Therefore, a total of 44 health facilities offering RCH services located in the Mbeya region were visited and surveyed.

The second step involved the selection of pregnant women for each selected health facility. An eligibility form was used to list all pregnant women attending RCH services in the selected health facility. The resulting list of pregnant mothers served as the sampling frame for the selection of participants who met the inclusion criteria. Systematic Random Sampling was then carried out by using the list of mothers to randomly select required pregnant women for each facility to participate in the survey based on probability and proportion to size sampling for the specific facility.

### Data collection

Data were collected through interviews guided by a structured questionnaire and, laboratory analysis of urine samples. A standard structured questionnaire was constructed in English and translated into Kiswahili, a language that is spoken by almost 95% of Tanzanians (see Extended data). ^24^ To ensure the quality of the translation, back-translation was performed by independent translators and reviewed by field staff in Mbeya. Pre-testing was done to evaluate the quality of the translations in terms of comprehensibility, readability, and relevance to assess face validity.

The interviews were administered by a trained Nurse Midwife in face-to-face interviews with participants, before the collection of urine samples. Initial interviews were administrated to determine various social demographic characteristics and dietary factors concerning iodine status, including participant’s age, marital status, education, household assets possessions, socioeconomic status, parity, stage of pregnancy, and dietary habits.

### Urine sample collection and laboratory analysis

A trained Nurse Midwife collected urine samples from consented study participants. The urine samples were collected in a disposable plastic screw caped container of 100 ml. Before urine collection, the approximate volume of urine sample required was pre-marked by a trained Nurse Midwife on urine containers and instructed the participant on collecting her urine in the container. The collected urine sample was stored in the cool-box with a temperature of approximately 2-8 ^o^C for 2-4hours. At the temporary laboratory, the sample was processed, transferred into two 2 ml vials, and then labeled by the trained laboratory technician. The sample into vials was kept stored at -20 ^o^C. All urine samples were shifted to TFNC laboratory (located in Dar es Salaam) for further analysis within one month. The urine samples were analyzed using the ammonium persulfate digestion method, as previously described by Sandell-Kolthoff reaction. ^25^ TFNC laboratory is registered and successfully participated in the quality assurance program for Ensuring the Quality of Urinary Iodine Procedures (EQUIP) ^26^ offered by the Centres for Disease Control and Prevention (CDC), Atlanta, Georgia, USA. The assay accuracy was assessed using reference quality-control urine specimens obtained from the CDC. The assay detection limit was <5.0 μg/L with the coefficient of variation <10%, when compared to the reference method. ^26^

### Variables

Outcome/response variable

Median UCI as a response variable was split into three categories as per WHO recommended level of iodine micronutrient. The median urine iodine concentration (mUIC) indicated the level of iodine in urine (μg/L) (see Underlying data). ^24^

UIC 1 (Iodine <150 μg/L) = Insufficient iodine

UIC 2 (150< Iodine <249 μg/L) = Sufficient iodine

UIC 3 (Iodine >250 μg/L) = Above WHO recommended/excessive iodine intake

Independent variables/predictors

The study includes a set of independent variables to understand the extent and variations between the levels of iodine micronutrients among the participants. Socio-demographic variables assessed included age, residency (district), education level, occupation status, number of pregnancies, visits to the ANC and, upper mid-arm circumference (MUAC), which is the most accurate way to measure fat-free mass outside of a laboratory. Household wealth was also assessed. To do so, durable household assets that indicate wealth such as a radio, television, and telephone were recorded as (1) “available and in working condition” or (0) “not available and/or not in working condition.” Principal component analysis, PCA was then conducted to categorize households into five quartiles of wealth, with 1 being the lowest and 3 the highest. Diet, in specific consumption of certain foods, such as fish, dairy products and processed meat and, refined and baked foods was also assessed among the participants, using 24-hour recalls.

### Data analysis

The data were analyzed using Stata v 15.1(RRID: SCR_012763). Stata is proprietary software but an open-access alternative in which the sequence could have been generated is Microsoft Excel (RRID: SCR_016137). Descriptive statistics were used to summarize the data of study participants. Pearson’s chi-square test and *p*-values were used to test for the significance of each of the potential risk factors in bivariate analysis. Multinomial logistic regression models were used to adjust for cofounders and predict the true association between the dependent and independent variables. All tests were two-tailed, and the significance level was set at p ≤ 0.05.

### Ethical approval and informed consent

Ethical clearance was obtained from the National Institute for Medical Research (NIMR) with reference number NIMR/HQ/R.8a/Vol. IX/2589 and appropriate authorization was given from the Regional, Council and health facility level. All eligible subjects were given information about the survey and were asked to sign a written informed consent form before participation.

## Results

### Descriptive of the study participants

The socio-demographic profile of overall sample is shown in Table 1. In this study, 420 agreed to participate (response rate of 73%). More than half of the respondents belonged to age 15-24 years. The mean age of the pregnant women was 25.49 (± 6.37) years. The majority of the respondents (70%) had primary education, two third of the respondents has been pregnant more than once. The household socio-economic composition of the sample shows a better distribution of all categories of respondents with about one third belong to the poorest quintile. More than two third of the respondents were self-employee (84.5%) followed by not employed (11.9%), and formal employee (3.6%). Improved source of water was reported by 71% of the participants. The distribution of respondents according to dietary habit, more than half of the respondents were reported that they consumed fish (68%) and, more than 90% consumed dairy products.

**Table 1.** Frequency distribution of the study participant in Mbeya (n = 420).

Variables	Category	n	%
Age group	15-24	215	52.18
	25-34	147	35.68
	35-49	50	12.14
Education level	No formal education	34	8.10
	Primary education	301	71.67
	Secondary and above	85	20.24
Marital status]	Married	238	56.67
	Cohabit	133	31.67
	Single	39	9.29
	Divorced	10	2.38
Occupational status	Formal employment	15	3.57
	Self-employment	355	84.52
	Not employed	50	11.90
Antenatal care center (ANC) visit	1 visit	163	38.81
	2-3 visits	226	53.81
	More than 3 visits	31	7.38
Residence	Chunya District Council	45	10.71
	Mbeya District Council	97	23.10
	Mbarali District Council	93	22.14
	Kyela District Council	50	11.90
	Rungwe District Council	68	16.19
	Busokelo District Council	33	7.86
	Mbeya City	34	8.10
Number of pregnancies	Primiglavida	104	24.76
	Multiglavida	316	75.24
Type of water source	Improved	302	71.90
	Unimproved	118	28.10
Mid-upper arm circumference (MUAC) categorization	MUAC < 23 cm	16	3.81
	MUAC ≥ 23 cm-MUAC < 33 cm	383	90.19
	MUAC ≥ 33 cm	21	5.0
Consumption of fish	No	287	68.3
	Yes	133	31.7
Consumption of Dairy products	No	381	90.7
	Yes	39	9.8
Consumption of Processed meat	No	410	97.6
	Yes	10	2.4
Urinary Iodine Concentration (UIC) categorization	Insufficient (UIC 0–149 μg/l)	72	17.14
	Sufficient (UIC 150–249 μg/l)	102	24.29
	Above recommended (>250 μg/l)	246	58.57

### Urinary iodine concentration (UIC)

The median UIC in the present study was 279.4μg/L, and it ranged from 26.1-1915μg/L. According to the UIC results, 17.14% of participants had an insufficient iodine intake, 24.29% had sufficient urine iodine concentration, and 58.57% had above the WHO recommended level of iodine in urine ( Table 1).

### Bivariate analysis

Table 2 presents a cross-tabulation of the median UIC status (mUIC) and socio-demographic, economic and dietary factors among pregnant women in Mbeya. Of 215 participants aged between 15-24 years, 17% had UIC (0–149 μg/l) that would be considered insufficient, and 55.8% had UIC (>250 μg/l) above the WHO recommended levels. The residence profile of the sample shows that, Chunya and Mbarali DCs have the highest percentage (above 70%) of the WHO recommended UIC among the pregnant women in Mbeya. On other hand, Rungwe DC had the highest percentage (27.9%) of participants with insufficient urine iodine concentrations. From the 133 participants who had fish in their diet, UIC was insufficient in 23%, sufficient in 19.4%, and 56.9% had above the WHO recommended level.

**Table 2.** Predictors of median urine iodine concentrations (mUIC) among pregnant women in Mbeya (n = 420).

Variable	Category	Insufficient (Urinary Iodine Concentration (UIC) 0–149 μg/l)		sufficient (UIC 150–249 μg/l)		Above recommended (>250 μg/l)		Chi-square	P-value
		%	n	%	n	%	N		
Age group	15-24	17.2	37	26.9	58	55.8	120	4.0208	0.403
	25-34	16.3	24	22.4	33	61.2	90		
	35-49	14.0	7	16.0	8	70.0	35		
Education level	No formal education	26.4	9	17.6	6	55.8	19	4.314	0.634
	Primary education	15.6	47	24.9	75	59.4	179		
	Secondary and above	18.8	16	24.7	21	56.5	48		
Wealth Index	1 tercile	20.7	29	26.4	37	52.8	74	4.7325	0.316
	2 tercile	15.7	22	20.0	28	64.2	90		
	3 tercile	15.0	21	26.4	37	58.5	82		
Marital status	Married	18.4	44	22.2	53	59.2	141	2.71	0.838
	Cohabit	15.0	20	28.5	38	56.3	75		
	Single	17.9	7	23.0	9	58.9	23		
	Divorced	10.0	1	20.0	2	70.0	7		
Occupational status	Formal employment	20.0	3	33.3	5	46.6	7	4.132	0.388
	Self-employment	17.4	62	22.5	80	60.0	213		
	Not employed	14.0	7	34.0	17	52.0	26		
Antenatal care center (ANC) visit	1 visit	17.1	28	23.9	39	58.9	96	3.3699	0.498
	2-3 visits	18.5	42	24.7	56	56.6	128		
	More than 3 visits	6.4	2	22.5	7	70.9	22		
Residence	Chunya DC	6.6	3	22.2	10	71.1	32	31.987	0.001 ^*^
	Mbeya DC	21.6	21	32.9	32	45.3	44		
	Mbarali DC	11.8	11	13.9	13	74.1	69		
	Kyela DC	12.0	6	20.0	10	68.0	34		
	Rungwe DC	27.9	19	25.0	19	47.0	32		
	Busokelo DC	24.2	8	27.2	9	48.4	16		
	Mbeya city	11.7	4	32.3	11	55.8	19		
Number of pregnancies	Primiglavida	16.3	17	22.1	23	61.5	64	0.527	0.768
	Multiglavida	17.7	55	25.0	79	57.5	182		
Type of water source	Improved	18.5	56	23.5	71	57.9	175	0.567	0.457
	Unemployed	13.5	16	26.2	31	60.1	71		
Mean- upper arm circumference (MUAC) categorization	MUAC < 23 cm	12.5	2	18.7	3	68.7	11	0.987	0.912
	MUAC ≥ 23 cm-MUAC < 33 cm	17.4	67	24.2	91	58.2	223		
	MUAC ≥ 33 cm	14.2	3	28.5	6	57.1	12		
Consumption of fish	No	13.7	38	26.8	74	59.4	164	7.5619	0.023 ^*^
	Yes	23.6	34	19.4	28	56.9	82		
Consumption of Dairy products	No	17.1	60	23.7	83	59.0	206	0.2912	0.865
	Yes	16.9	12	26.7	16	56.3	40		
Consumption of Processed meat	No	17.2	71	23.8	98	58.8	242	2.0475	0.359
	Yes	11.1	1	44.4	4	44.4	4		
Consumption of refined and baked	No	18.9	14	24.3	18	56.7	42	0.2158	0.898
	Yes	16.7	58	24.2	84	58.9	204		

* Represents p value: p < 0.05.

### Multivariate analysis

The multivariate analysis are presented in Tables 3a and 3b. The chi-square model (63.51) was 0.0176, with p < 0.05.

**Table 3a.** Multivariate analysis on factors associated with values below the WHO recommendation for mUIC among pregnant women in Mbeya (n = 420).

Independent			95% confidence interval for OR		
Variable	Category	OR	Lower bound	Upper bound	P-value
Consumption of fish	No	1			
	Yes	2.60	1.31	5.15	0.006 *
Consumption of Dairy products	No	1			
	Yes	0.96	0.41	2.28	0.940
Consumption of Processed meat	No	1			
	Yes	0.32	0.03	3.19	0.334
Consumption of refined and baked	No	1			
	Yes	0.79	0.33	1.91	0.609
Residence	Mbeya district council (DC)	1			
	Chunya DC	0.38	0.08	1.65	0.199
	Mbarali DC	1.15	0.38	3.44	0.793
	Kyela DC	0.85	0.25	2.92	0.809
	Rungwe DC	2.43	0.95	6.19	0.061
	Busokelo DC	1.79	0.52	6.11	0.351
	Mbeya city	0.77	0.18	3.24	0.732
Age group	15-24	1			
	25-34	1.11	0.50	2.44	0.782
	35-49	1.45	0.42	4.98	0.553
Wealth Index	1 quantile	1			
	2 quantile	1.62	0.62	4.26	0.321
	3 quantile	1.28	0.42	3.90	0.663
Education level	No formal education	1			
	Primary education	0.29	0.08	0.99	0.049 *
	Secondary and above				
Mean upper arm circumference (MUAC) categorization	MUAC < 23 cm	1			
	MUAC ≥ 23 cm-MUAC < 33 cm	1.27	0.18	8.90	0.807
	MUAC ≥ 33 cm	1.17	0.10	13.31	0.896
Number of pregnancies	Primiglavida	1			
	Multiglavida	0.83	0.34	2.01	0.683

* Represents p value: p < 0.05.

**Table 3b.** Multivariate analysis on factors associated with values above the WHO recommendation for mUIC among pregnant women in Mbeya (n = 420).

Independent			95% confidence interval for OR		
Variable	Category	OR	Lower bound	Upper bound	P-value
Consumption of fish	No	1			
	Yes	1.24	0.71	2.15	0.438
Consumption of Dairy products	No	1			
	Yes	0.90	0.46	1.73	0.754
Consumption of Processed meat	No	1			
	Yes	0.50	0.11	2.31	0.379
Consumption of refined and baked	No	1			
	Yes	0.99	0.50	1.96	0.998
Residence	Mbeya DC	1			
	Chunya DC	2.05	0.84	4.96	0.110
	Mbarali DC	4.09	1.85	9.01	0.000 *
	Kyela DC	2.15	0.88	5.23	0.089
	Rungwe DC	1.49	0.67	3.29	0.321
	Busokelo DC	1.55	0.56	4.26	0.390
	Mbeya city	1.45	0.54	3.94	0.456
Age group	15-24	1			
	25-34	1.47	0.81	2.69	0.201
	35-49	2.51	0.99	6.33	0.050 *
Wealth Index	1 tercile	1			
	2 tercile	1.41	0.66	3.03	0.367
	3 tercile	2.08	0.91	4.71	0.079
Education level	No formal education	1			
	Primary education	1.04	0.37	2.94	0.929
	Secondary and above	1.18	0.35	3.95	0.777
MUAC categorization	MUAC < 23 cm	1			
	MUAC ≥ 23 cm-MUAC < 33 cm	1.01	0.25	4.05	0.985
	MUAC ≥ 33 cm	0.74	0.13	4.27	0.745
Number of pregnancies	Primiglavida	1			
	Multiglavida	0.64	0.32	1.26	0.203

* Represents p value: p < 0.05.

Table 3a presents a multivariate analysis of the factors related to the group of women who are below the WHO recommendation for mUIC among pregnant women. After taking into account confounding factors, it was found that dietary and socio-demographic factors were significantly associated with the low WHO recommendation for mUIC among pregnant women. The analysis showed that pregnant women who consume fish were more likely to have a low WHO recommendation for mUIC, with an Adjusted OR of 2.60 (95% CI 1.31-5.15). In contrast, pregnant women who had at least primary education were less likely to have a low WHO recommendation for mUIC, with an Adjusted OR of 0.29 (95% CI 0.08-0.99). Table 3a presents a multivariate analysis of the factors related to the low WHO recommendation for mUIC among pregnant women. After taking into account confounding factors, it was found that dietary and socio-demographic factors were significantly associated with the low WHO recommendation for mUIC among pregnant women. The analysis showed that pregnant women who consume fish were more likely to have a low WHO recommendation for mUIC, with an Adjusted OR of 2.60 (95% CI 1.31-5.15). In contrast, pregnant women who had at least primary education were less likely to have a low WHO recommendation for mUIC, with an Adjusted OR of 0.29 (95% CI 0.08-0.99).

Table 3b presents a multivariate analysis of the factors related to the group of women who are above the WHO recommendation for mUIC among pregnant women. After taking into account confounding factors, it was found that only socio-demographic factors were significantly associated with the above WHO recommendation for mUIC among pregnant women. Thus, pregnant women resident in Mbarali DC, aged between 35-49 years and belonging to the highest socio-economic status [Adjusted OR = 4.09 (95% CI 1.85-9.010], [Adjusted OR = 2.51 (95% CI 0.99-6.330] and [Adjusted OR = 2.08 (95% CI 0.91-4.71) respectively were at greatest risk of excess mUIC.

## Discussion

This is the first population-based cross-sectional study to assess the magnitude of iodine status and the association with socio-demographic factors and diet in Tanzanian pregnant women. The findings of the study are important since iodine insufficiency is the most prevalent micronutrient insufficiency, affecting 28.9% of the world population, ^27^ particularly affecting women living in developing countries. ^28^ Iodine deficiency disorders in Tanzania are high with the most recent figures indicating that more than 40% of the population in the country lives in geographical regions prone to iodine insufficiency. ^6^ However, this data is largely outdated, as more recent data as well as the most recent efforts to reduce iodine insufficiency have focused on primary school children in Tanzania, ^6^ whilst the iodine micronutrient status among pregnant women has been limited in recent years. A reanalysis of the 2010 Tanzania demographic and health survey reported that 54% of pregnant women had subclinical iodine deficiency. ^19^

The present research looks for potential socio-demographic and dietary factors associated with levels of mUIC both below and above WHO recommendations. Our study found that residence in Mbarali district, age between 35-49 years, and belonging to high socio-economic status were associated with an increasing odds of pregnant women having excess mUIC levels. This could be explained by the fact that the Mbarali district is home to the Ruaha National Park, which attracts food products to its business district that are preserved by iodized salt. As previous documented ^29^^,^^30^ the frequency and intake of food products preserved or snacks sprinkled with iodated salt were one of the four scenarios of excessive iodine intakes, the other three were: close to salt factories; losses less than expected because of not passing through all the steps in the salt marketing chain, and districts close to large salt processing factories adhering to USI. Thus, it is important to continue monitoring the distribution, packaging and handling iodated salt and, similarly to monitor thyroid function and its associated disorders in this population since excessive iodine is thought to matter most at the time of fetal development.

This study also looked for evidence of the factors likely to be influencing the prevalence of insufficient mUIC among pregnant women in Mbeya. We found that consumption of fish was associated with lower mUIC. This finding could be explained by the fact that iodine levels in freshwater fish may be low and they are not preserved with salt. ^6^ The poor iodation technologies and supply of potassium iodate in many small and medium salt producers could be the reason behind insufficient iodine observed in this survey. ^31^^,^^32^ Moreover, during pregnancy there are variations in the functionality of the thyroid. This can increase the risk of insufficient iodine intake for some mothers. As such, predicting UIC based on usage of iodized salt alone, may not be accurate. ^33^^–^^35^ However, other studies have documented that freshwater fish may contain Iodine at levels that can improve daily Iodine intake. ^31^

In countries with successful USI programs, ^36^^,^^37^ studies have reported an optimal median UIC in pregnant women in comparison to Tanzania. ^20^^,^^38^ As such, USI remains the most cost-effective strategy for achieving reduced IDD. However, the full implementation of USI remains a challenge in many sub–Saharan African countries including Tanzania, ^38^ largely due to the lack of adequate enforcement and, the inadequate monitoring of small-scale salt producers who often do not comply with USI legislation. ^6^

This analysis also indicated that pregnant women who had a primary school education were at lower risk of iodine insufficiency; however, further studies are needed to investigate this association. ^30^ Similar evidence has been reported in previous studies among primary school children in Tanzania alerts policymakers to consider adjusting the amount of iodine added to salt along with the obligation of reducing discretionary foods and salt intake. ^39^^,^^40^

WHO recommended an increased iodine intake for pregnant women, although evidence is weak. ^41^ Indeed, detrimental effects from more than adequate and excessive iodine intake have been reported in general populations. ^42^^–^^44^ Shi et al. have reported on the associations between UIC and thyroid health among pregnant women and recommend a lower limit for maternal iodine intake during pregnancy than that currently advised by the WHO. ^45^ This is also an area in need of further investigation. The question remains how much iodized salt pregnant women in Mbarali district should consume, and at what concentration. As our findings illustrate, it is extremely difficult for USI to avoid not only deficiency but also excess, especially in mixed urban and rural settings in areas with complex salt production by small and large producers. ^6^^,^^18^

The strength of this study is in the fact that its large population-based sample size managed to demonstrate important factors that could explain factors associated with both excessive and insufficient iodine among pregnant women. However, there were limitations as follows: first, the use of UIC to determine individual iodine status could be limited due to the potential for misclassification of participants because of day-to-day variations. Second, UIC reflects recent iodine intake or exposure rather than chronic individual iodine status. Third, the use of iodized salt was not assessed in this study. Finally, it would have been useful to have a non-pregnant control group to help ascertain whether lower mean UIC concentrations during pregnancy could be attributed to pregnancy itself or the diet.

## Conclusion

This study demonstrated a significant association between geographical factors (residence in the Mbarali district) and median urine iodine concentration above WHO recommended. Further, this study found association between inadequate iodine in diet and insufficient median urine iodine concentration as indicated by the World Health Organization recommendation. Therefore, educational programs on iodine intake should be strengthen as attending at least primary education was found to be a protective factor for insufficient median urine iodine concentration. This study also recommends further longitudinal studies. Further, attending at least primary education was found to be a protective factor for insufficient median urine iodine concentration. Controlling risk factors through strengthening the USI program to include monitoring excessive iodine exposures will reduce the detrimental effects of iodine during pregnancy. This study also recommend for further longitudinal studies. It illustrates how difficult it is to adjust salt iodation levels so as to avoid both deficiency and excess and the importance of continued monitoring and adjustment of iodation levels at regional and even local levels as needed.

## Data availability

### Underlying data

Open Science Framework (OSF): Factors associated with inadequate urinary iodine concentration among pregnant women in Mbeya region Tanzania. DOI: https://osf.io/7ysb9/. ^24^

This project contains the following underlying data:

• MBMNS_MUIC10082021: This is the SPSS database file that contained all the laboratory assessment variables for the medium urine iodine concentrations.

This project also contains the following extended data:

• Questionnaire English version: This file contains all the questions used to interview pregnant women in Mbeya.
• Questionnaire Swahili version: This file is the Swahili version of Questionnaire.

Data are available under the terms of the Creative Commons Zero “No rights reserved” data waiver (CC0 1.0 Public domain dedication).

## Author contributions

Conceptualization, TL, RM., AH. SEJ and GHL; project administration and resources, AS, RN, FK and GHL; formal analysis and writing—original draft, TL, RM, AH, SEJ, HAP, AS, RN, FK, ET and GHL; reviewed and edited the manuscript. GB and, RB. All authors: Reviewed and agreed upon the final manuscript.
