# Supplementary material for: Factors associated with inadequate urinary iodine concentration among pregnant women in Mbeya region Tanzania
Source: F1000Res. 2024 Aug 22;10:858. Originally published 2021 Aug 26. [Version 5] doi: 10.12688/f1000research.55269.5 (PMC11358688; doi:10.12688/f1000research.55269.5)
Supplement: Peer review history

# Reviewer report: Reviewer response for version 5

**DOI:** 10.5256/f1000research.163952.r316203

**Published:** 2024-08-28

**Authors:** Ted Greiner

**Affiliations:** 1 Member, Executive Committee; Editor-in-Chief of Association journal World Nutrition (https://worldnutritionjournal.org/index.php/wn), World Public Health Nutrition Association (Ringgold ID: 26716), London, UK

I approve this 5th version.

Is the work clearly and accurately presented and does it cite the current literature?

Partly

If applicable, is the statistical analysis and its interpretation appropriate?

Yes

Are all the source data underlying the results available to ensure full reproducibility?

Yes

Is the study design appropriate and is the work technically sound?

Yes

Are the conclusions drawn adequately supported by the results?

Partly

Are sufficient details of methods and analysis provided to allow replication by others?

Yes

Reviewer Expertise:

Public Health Nutrition

I confirm that I have read this submission and believe that I have an appropriate level of expertise to confirm that it is of an acceptable scientific standard.

# Reviewer report: Reviewer response for version 4

**DOI:** 10.5256/f1000research.163953.r270358

**Published:** 2024-06-17

**Authors:** Ted Greiner

**Affiliations:** 1 Member, Executive Committee; Editor-in-Chief of Association journal World Nutrition (https://worldnutritionjournal.org/index.php/wn), World Public Health Nutrition Association (Ringgold ID: 26716), London, UK

1. "Table 3a. Multivariate analysis on factors associated with the low WHO recommendation"

 Should end with "values below" or "women below" the WHO recommendation, since you are not commenting on the recommendation itself. The same is true for Table 3b.

 Similarly, below the tables, " factors related to the low WHO" should be something like "factors related to the group of women who are below the WHO" and similar for the next paragraph regarding those above the recommendation. The paragraph about 3a is repeated a second time.

 2. You write that ref. 19 found "54% of pregnant women with iodine deficiency" but the title of that article should be repeated (subclinical). If this is based on goiter rather than UIC, I suggest mentioning this because goiter in Tanzanian adults is likely related more to past than present IDD. Is it correct that this analysis was done in 2010? (It was published in 2017.) Rather than saying recent attention to pregnant women has been overlooked, I suggest saying it has been limited. 

 3. "distract" should presumably be district.

 4. After saying "In countries with successful USI programs, studies have reported an optimal median UIC in pregnant women." I suggest giving ref. 37 (which is incomplete) if it says that. Then, in comparison, give the median from your study. 

 5. In the conclusions, the first mention of "This study also recommend for further longitudinal studies." should be deleted. Instead of "recommend for", I suggest "recommends".

Is the work clearly and accurately presented and does it cite the current literature?

Partly

If applicable, is the statistical analysis and its interpretation appropriate?

Yes

Are all the source data underlying the results available to ensure full reproducibility?

Yes

Is the study design appropriate and is the work technically sound?

Yes

Are the conclusions drawn adequately supported by the results?

Partly

Are sufficient details of methods and analysis provided to allow replication by others?

Yes

Reviewer Expertise:

Public Health Nutrition

I confirm that I have read this submission and believe that I have an appropriate level of expertise to confirm that it is of an acceptable scientific standard, however I have significant reservations, as outlined above.

# Response

**Published:** 2024-07-31

**Authors:** Ray Masumo

**Affiliations:** Tanzania Food and Nutrition Centre, Tanzania

We appreciate the time and effort that you and the reviewers dedicated to providing valuable feedback on our manuscript.

 Thank you very much.

 Here is a point-by-point response to the reviewers’ comments and concerns.

**Results**

**Comment 1:** *"Table 3a. Multivariate analysis on factors associated with the low WHO recommendation" Should end with "values below" or "women below" the WHO recommendation, since you are not commenting on the recommendation itself. The same is true for Table 3b.*

*Similarly, below the tables, “factors related to the low WHO" should be something like "factors related to the group of women who are below the WHO" and similar for the next paragraph regarding those above the recommendation. The paragraph about 3a is repeated a second time.*

**Response**: We agree with this comment and have included your suggestion.

**Discussion:**

**Comment 2**: *You write that ref. 19 found "54% of pregnant women with iodine deficiency" but the title of that article should be repeated (subclinical). If this is based on goiter rather than UIC, I suggest mentioning this because goiter in Tanzanian adults is likely related more to past than present IDD. Is it correct that this analysis was done in 2010? (It was published in 2017.) Rather than saying recent attention to pregnant women has been overlooked, I suggest saying it has been limited*.

**Response**: We agree with this comment and have rewritten the discussion section to stipulate that 54% of pregnant women had subclinical iodine deficiency.  

**Comment 3**: *"distract" should presumably be district.*

**Response**: Thank you so much for pointing this out and, we have corrected it.

**Comment 4**: *After saying "In countries with successful USI programs, studies have reported an optimal median UIC in pregnant women." I suggest giving ref. 37 (which is incomplete) if it says that. Then, in comparison, give the median from your study.*

**Response**: We agree with this comment and have rewritten the paragraph as suggested.

**Conclusion:**

**Comment 5**: *In the conclusions, the first mention of "This study also recommend for further longitudinal studies." should be deleted. Instead of "recommend for", I suggest "recommends".*

**Response**: Thank you for pointing this out. We agree with this comment and edited the sentence as suggested.

# Reviewer report: Reviewer response for version 2

**DOI:** 10.5256/f1000research.137830.r166505

**Published:** 2023-04-05

**Authors:** Ted Greiner

**Affiliations:** 1 Member, Executive Committee; Editor-in-Chief of Association journal World Nutrition (https://worldnutritionjournal.org/index.php/wn), World Public Health Nutrition Association (Ringgold ID: 26716), London, UK

Factors associated with inadequate urinary iodine concentration among pregnant women in Mbeya region Tanzania

 This is a useful, reasonably well-done study on an important issue. The OR data referred to in the discussion and any other OR data that was analyzed need to be presented in the Results section. The authors write well in English but it still needs to be proofread and several further corrections made in addition to the comments below:

1. I think it would be useful to give some of the early provincial school-based survey data from Tanzania showing that Mbeya had one of the highest prevalences of goiter, thus indicating the value of having chosen it for this study.
2. Is there no more modern reference than your #5? Its data are over 30 years old.
3. “Although USI is arguably the successful [health] intervention in global history”
4. “UNICEF documented that [in] many African countries ”
5.  “whose diets contain sufficient [should presumably read “substantial”] amounts of iodine-depleting foods”
6. “more than 10% of Tanzania households [which] remain at risk”
7. “It is also necessary to set upper as well as lower limits for maternal iodine intake to ensure optimal health outcomes are achieved” - This does not seem to be a task related to the present paper. On the other hand, its findings could be useful in future decision making regarding whether levels of iodine added to fortified salt should be increased or decreased, especially if such decisions can be taken at regional or local salt facility levels.
8. “All pregnant women aged 15-49 years who attended [the selected RCH] clinics within their first and second trimesters ”
9. “420 agreed to take part, as per the calculated sample size” - The final clause in this statement is premature and in any case incorrect and needs to be reworded or deleted.
10. “medium urine iodine concentration” Presumably should be “median”?
11. “Median UCI as a response variable was split into three categories as per WHO recommended level of iodine micronutrient. A new variable called medium urine iodine concentration (UIC) was developed to indicate the level of iodine in urine (μg/L) (see Underlying data).21” - This is unclear and seems unnecessary. Surely each UIC was simply placed into one of the three existing WHO categories.
12. Is the wealth index in Table 1 really quintiles? If so, which one is completely absent? If it is terciles, which seems likely, remove it from the table, as 33% in each category is literally the definition of a tercile, not a finding.
13. In Table 2, the n’s are missing from secondary education.
14. I don’t see the point in Table 3. It cannot be interpreted on its own. It does not even mention fish. Seeing it does not add any information or clarity to what is presented in the text and it only includes a few values, which could be given in the text if important.
15. “This research looks for evidence of the factors influence excess of UIC above the WHO recommended level.” Suggestion: “evidence of the factors likely to be influencing the prevalence of excess UIC”
16. “Geographical factors i.e. residence in Mbarali district, and the aged between 35-49 years were play an important role in increasing the risk of pregnant women having excess UIC above the WHO recommended level: [Adjusted OR = 4.09 (95% CI 1.85-9.01)] and, [Adjusted OR = 2.51 (95% CI 0.99-6.33)] respectively.” - This is confusing. It is written as if it’s evidence from the present study, but this was not included in the results section. Then it is referenced as if it is Assey’s finding. The next sentence (which is the one that presumably should be cited for Assey) is also confusing. Why would being closed to earlier access to iodated salt be associated with increased risk of excess?
17. In this sentence: “The poor iodation technologies and supply of potassium iodate in many small and medium salt producers could be the reason behind consumption of freshwater fish and insufficient iodine observed in this survey” I suggest deleting “consumption of freshwater fish and” since it is illogical.
18. “This analysis also indicated that pregnant women who had a primary school education were at lower risk of iodine insufficiency [Adjusted OR = 0.29 (95% CI 0.08-0.99)], ” was also not presented in the results section.

Is the work clearly and accurately presented and does it cite the current literature?

Partly

If applicable, is the statistical analysis and its interpretation appropriate?

Yes

Are all the source data underlying the results available to ensure full reproducibility?

Yes

Is the study design appropriate and is the work technically sound?

Yes

Are the conclusions drawn adequately supported by the results?

Partly

Are sufficient details of methods and analysis provided to allow replication by others?

Yes

Reviewer Expertise:

My areas of expertise are infant feeding and combatting micronutrient deficiencies (A, Fe, I) at public health levels. I am co-author on several publications related to IDD in Tanzania and worked on the issue some in the field there, for example a school goiter survey. I was involved with UIC measurement surveys, but am not myself expert on nor have I ever personally utilized laboratory methods in research.

I confirm that I have read this submission and believe that I have an appropriate level of expertise to confirm that it is of an acceptable scientific standard, however I have significant reservations, as outlined above.

# Response

**Published:** 2023-04-22

**Authors:** Ray Masumo

**Affiliations:** Tanzania Food and Nutrition Centre, Tanzania

We appreciate the time and effort that you have dedicated to providing constructive feedback on our manuscript entitled ‘Factors associated with inadequate urinary iodine concentration among pregnant women in Mbeya region Tanzania’.

 Thank you very much.

 Here is a point-by-point response to the reviewers’ comments and concerns.

**General comment:**

**Comment 1:** *This is a useful, reasonably well-done study on an important issue. The OR data referred to in the discussion and any other OR data that was analyzed need to be presented in the Results section.*

**Response**: Thank you very much for pointing this out and, we have incorporated your suggestion throughout the manuscript.

**Specific comments:**

**Comment 1:** *I think it would be useful to give some of the early provincial school-based survey data from Tanzania showing that Mbeya had one of the highest prevalences of goiter, thus indicating the value of having chosen it for this study.*

**Response:** We agree with this comment and have added more information on the early provincial school-based survey data from Tanzania showing that Mbeya had one of the highest prevalence of goiter.

**Comment 2:** *Is there no more modern reference than your #5? Its data are over 30 years old.*

**Response**: Thanks for pointing this out. We have replaced reference #5 with the recent reference.

**Comment 3**: *“Although USI is arguably the successful [health] intervention in global history”*

**Response: **Thank you very much for pointing this out and we have incorporated your suggestion.

**Comment 4:** *“UNICEF documented that [in] many African countries”*

**Response:** We agree with this comment and incorporated the suggestion.

**Comment 5**: *“whose diets contain sufficient [should presumably read “substantial”] amounts of iodine-depleting foods”*

**Response**: We agree with this comment and replaced the word ‘sufficient’ as suggested.

**Comment 6**: *“More than 10% of Tanzania households [which] remain at risk”*

**Response**: Thank you very much for pointing this out and, we have incorporated your suggestion.

**Comment 7:** *“It is also necessary to set upper as well as lower limits for maternal iodine intake to ensure optimal health outcomes are achieved” - This does not seem to be a task related to the present paper. On the other hand, its findings could be useful in future decision-making regarding whether levels of iodine added to fortified salt should be increased or decreased, especially if such decisions can be taken at regional or local salt facility levels*

**Response:** Thank you for pointing this out and we have rephrased the statement for clarity.

**Comment 8** *: “All pregnant women aged 15-49 years who attended [the selected RCH] clinics within their first and second trimesters”*

**Response**: We agree with this comment and have incorporated your suggestion.

**Comment 9** *:* *“420 agreed to take part, as per the calculated sample size” - The final clause in this statement is premature and in any case incorrect and needs to be reworded or deleted* *.*

**Response**: Thank you for this suggestion and we have incorporated your suggestion.

**Comment 10**: *“medium urine iodine concentration” Presumably should be “median”?*

**Response**: We agree with this comment and make the correction as suggested 

**Comment 11**: *“Median UCI as a response variable was split into three categories as per WHO recommended level of iodine micronutrient. A new variable called medium urine iodine concentration (UIC) was developed to indicate the level of iodine in urine (μg/L) (see Underlying data).21” - This is unclear and seems unnecessary. Surely each UIC was simply placed into one of the three existing WHO categories*.

**Response**: We highly appreciate the reviewers’ insightful and helpful comments on our manuscript. We have rewritten the statement for clarity.

**Comment 12**: *Is the wealth index in Table 1 really quintiles? If so, which one is completely absent? If it is terciles, which seems likely, remove it from the table, as 33% in each category is literally the definition of a tercile, not a finding.*

**Response**: We agree with this comment and make the correction as suggested.

**Comment 13**: *In Table 2, the n’s are missing from secondary education*

**Response**: Thank you for pointing that out, and we added the missing n’s and percentages

**Comment 14**: *I don’t see the point in Table 3. It cannot be interpreted on its own. It does not even mention fish. Seeing it does not add any information or clarity to what is presented in the text and it only includes a few values, which could be given in the text if important.*

**Response**: We agree with this comment and re-write the description of Table 3.

**Comment 15**: *“This research looks for evidence of the factors influence excess of UIC above the WHO recommended level.” Suggestion: “evidence of the factors likely to be influencing the prevalence of excess UIC”*

**Response**: We agree with this comment and incorporated your suggestion.

**Comment 16**: *“Geographical factors i.e. residence in Mbarali district, and the aged between 35-49 years were play an important role in increasing the risk of pregnant women having excess UIC above the WHO recommended level: [Adjusted OR = 4.09 (95% CI 1.85-9.01)] and, [Adjusted OR = 2.51 (95% CI 0.99-6.33)] respectively.” - This is confusing. It is written as if its evidence from the present study, but this was not included in the results section. Then it is referenced as if it is Assey’s finding. The next sentence (which is the one that presumably should be cited for Assey) is also confusing. Why would being closed to earlier access to iodated salt be associated with increased risk of excess?*

**Response**: We agreed with the comment and we have re-write the paragraph for clarity.

**Comment 17:** *In this sentence: “The poor iodation technologies and supply of potassium iodate in many small and medium salt producers could be the reason behind consumption of freshwater fish and insufficient iodine observed in this survey” I suggest deleting “consumption of freshwater fish and” since it is illogical.*

**Response:** We agree with this comment and incorporate the suggestion.

**Comment 18** *: “This analysis also indicated that pregnant women who had a primary school education were at lower risk of iodine insufficiency [Adjusted OR = 0.29 (95% CI 0.08-0.99)],” was also not presented in the results section.*

**Response**: Thank you for pointing that out, and we have re-write the paragraph for clarity.

# Reviewer report: Reviewer response for version 2

**DOI:** 10.5256/f1000research.137830.r150166

**Published:** 2022-09-22

**Authors:** Joyce Kinabo

**Affiliations:** 1 Department of Human Nutrition and Consumer Sciences, Sokoine University of Agriculture, Morogoro, Tanzania

Urine samples

- please indicate time of the day the samples were collected. Was it morning urine or random (i.e. collected at any time of the day)?

Variables

- 'Median UCI' - misspelt 

Is the work clearly and accurately presented and does it cite the current literature?

Partly

If applicable, is the statistical analysis and its interpretation appropriate?

Partly

Are all the source data underlying the results available to ensure full reproducibility?

Partly

Is the study design appropriate and is the work technically sound?

Partly

Are the conclusions drawn adequately supported by the results?

Partly

Are sufficient details of methods and analysis provided to allow replication by others?

Partly

Reviewer Expertise:

NA

I confirm that I have read this submission and believe that I have an appropriate level of expertise to confirm that it is of an acceptable scientific standard.

# Reviewer report: Reviewer response for version 1

**DOI:** 10.5256/f1000research.58830.r141642

**Published:** 2022-07-25

**Authors:** Joyce Kinabo

**Affiliations:** 1 Department of Human Nutrition and Consumer Sciences, Sokoine University of Agriculture, Morogoro, Tanzania

Abstract:

1. Use of the word 'insufficient' should be consistent through out the article.
2. Conclusion does not state explicitly the factors associated with insufficient/sufficient UIC.

Introduction:

1. Provide explanation as to why highland regions have highest prevalence of IDD considering that Tanzania has been implementing salt iodisation programmes for many years?

Method:

1. Change 'Ante-natal Care Clinics' to 'Reproductive and Health Clinics' and use it consistently through out the document.
2. Explain adequately how sampling procedure was carried out.
3. Was the questionnaire pre-tested before translation?
4. "interviews were done by health professional" - which cadre?
5. Provide more information about the conditions for storage of the urinary samples in the field, transportation and storage at the laboratory before analysis and for how long were the samples stored before analysis. What distance from the field to the laboratory? This is important because iodine in urine is know to undergo changes after some time.
6. Was their any effort to ensure that women at various stages of pregnancy were well represented in the sample? It will also be useful to know of the sample size recruited, what was the stage of pregnancy for each participant? This information is missing.
7. Why Mbeya region? Provide justification.

Results:

1. Area of residence seemed to have an influence on iodine status of pregnant women, it was expected to see a good description of the study areas in this manuscript. The information is missing. Could the authors strive to provide this information?
2. It will also be useful to know the stage of pregnancy (1st , 2nd, 3rd, trimester) of participants and if there were any association/interaction between area of residence, other factors and UIC.
3. Presentation of Tables should be improved; instead of brackets, authors could include columns for easy of reading.
4. What was the consumption of iodised salt? This could be one of the contributing factors to high levels of UIC observed in some areas of the study. But also consumption of foods especially vegetables of the brassica group that are known to have compounds that interact with iodine in the gut.
5. Clarification about consumption of processed meat.
6. Clearly stipulate factors that are associated with UIC in this section. Factors are not very clear.

Discussion:

1. How were the identified factors influenced iodine status? These have not been adequately articulated in this manuscript.
2. Was the use of iodised salt investigated in the present study? What is the level of consumption among pregnant women? What is the availability and accessibility to iodised salt in the study areas?
3. Authors indicated some strengths of the study; large sample size; but also identified limitations? One of the limitations was that they did not investigate use of iodised salt in the study area. This is a serious omission considering that iodised salt contributes to iodine intake and therefore UIC at least for a large part of the population. Additionally, there was no control group, which again raises concern over the design of this study.
4. Conclusion: It is a repeat of statement of results and not conclusion. Authors should look at the objectives of the study and provide a concrete conclusion as to whether the objectives were attained and what are the implications to individual women and society in general.

Is the work clearly and accurately presented and does it cite the current literature?

Partly

If applicable, is the statistical analysis and its interpretation appropriate?

Partly

Are all the source data underlying the results available to ensure full reproducibility?

Partly

Is the study design appropriate and is the work technically sound?

Partly

Are the conclusions drawn adequately supported by the results?

Partly

Are sufficient details of methods and analysis provided to allow replication by others?

Partly

Reviewer Expertise:

NA

I confirm that I have read this submission and believe that I have an appropriate level of expertise to confirm that it is of an acceptable scientific standard, however I have significant reservations, as outlined above.

# Response

**Published:** 2022-08-05

**Authors:** Ray Masumo

**Affiliations:** Tanzania Food and Nutrition Centre, Tanzania

We appreciate the time and effort that you and the reviewer have dedicated to providing valuable feedback on our manuscript. Thank you very much.

 Here is a point-by-point response to the reviewer's comments and concerns.

**Abstract**

**Comment 1: **The use *of the word 'insufficient' should be consistent throughout the article.*

**Response**: The word ‘insufficient’ instead of ‘deficiency’ will be used throughout the manuscript.

**Comment 2:** *Conclusion does not state explicitly the factors associated with insufficient/sufficient UIC*

**Response: **The conclusion will carefully be rephrased and show factors associated with insufficient/sufficient UIC.

**Introduction:**

**Comment 1:** *Provide an explanation as to why highland regions have highest prevalence of IDD considering that Tanzania has been implementing salt iodisation programs for many years?*

**Response:** The explanation has been provided on page 11, discussion section, paragraph 4 ‘ *However, the full implementation of USI remains a challenge in many sub–Saharan African countries including Tanzania, largely due to the lack of adequate enforcement and, the inadequate monitoring of small-scale salt producers who often do not comply with USI legislation*’.

**Method:**

**Comment 1:** *Change 'Ante-natal Care Clinics' to 'Reproductive and Health Clinics' and use it consistently throughout the document.*

**Response:** The word *'Ante-natal Care Clinics*' will replace *'Reproductive and Health Clinics*' throughout the document.

**Comment 2:** *Explain adequately how sampling procedure was carried out.*

**Response**: We agree, and the sampling procedures will be explained in more detail.

**Comment 3**: *Was the questionnaire pre-tested before translation?*

**Response: **Thank you very much for pointing this out. The questionnaire was pre-tested after translation.

**Comment 4:** *Interviews were done by health professional" - which cadre?*

**Response:** Interviews were done by trained Nurse Midwives (we will specify the cadre of health professionals).

**Comment 5**: *Provide more information about the conditions for storage of the urinary samples in the field, transportation, and storage at the laboratory before analysis and for how long were the samples stored before analysis. What distance from the field to the laboratory? This is important because iodine in urine is known to undergo changes after some time*.

**Response**: We agree - more information on the conditions of the urinary samples stored in the field, transportation, and storage at the laboratory before analysis will be added.

**Comment 6**: *Was their any effort to ensure that women at various stages of pregnancy were well represented in the sample? It will also be useful to know of the sample size recruited, what was the stage of pregnancy for each participant? This information is missing*.

**Response**: Thank you very much for pointing this out. The inclusion and exclusion criteria of this study were mentioned on page 4, paragraph 2 ‘ *Participants in their second trimester, a period during which fetal neurodevelopment is impacted by adequate maternal thyroid function, were included. To eliminate the effects of gestational age on thyroid hormone, participants beyond eight weeks of gestation were excluded*.

**Comment 7:** *Why Mbeya region? Provide justification.*

**Response:** This study is the part of IMAN project piloted in Mbeya and supported by the UNICEF-Tanzania and the Ministry of Health - Tanzania aims to demonstrate how the country can sustainably scale up delivery of a comprehensive package of interventions to improve maternal nutrition (Interventions, Platforms, and Enabling Environment/System strengthening).

**Results** :

**Comment 1**: *Area of residence seemed to have an influence on iodine status of pregnant women, it was expected to see a good description of the study areas in this manuscript. The information is missing. Could the authors strive to provide this information?*

**Response**: We agree and more information on the area of residence will be added.

**Comment 2**: *It will also be useful to know the stage of pregnancy (1st, 2nd, 3rd, trimester) of participants and if there were any association/interaction between area of residence, other factors and UIC.*

**Response**: The study participants were only pregnant women in the second trimester and the reason behind this was to avoid the effects of gestational age on thyroid hormone.

**Comment 3**: *Presentation of Tables should be improved; instead of brackets, authors could include columns for easy of reading*.

**Response**: We agree with this comment and will remove the bracket.

**Comment 4**: *What was the consumption of iodised salt? This could be one of the contributing factors to high levels of UIC observed in some areas of the study. But also consumption of foods especially vegetables of the brassica group that are known to have compounds that interact with iodine in the gut.*

**Response**: Consumption of iodized salt and vegetables of the *Brassica *group will be the limitation of the study.

**Comment 5**: *Clarification about consumption of processed meat*.

**Response**: Here we referred to meats that have been preserved by smoking, salting, curing, or addition of chemical preservatives. 

**Comment 6**: *Clearly stipulate factors that are associated with UIC in this section. Factors are not very clear.*

**Response**: We agree with this comment and will rephrase the paragraphs to stipulate clearly the factors associated with UIC.

**Discussion:**

**Comment 1**: *How were the identified factors influenced iodine status? These have not been adequately articulated in this manuscript*.

**Response**: We agree with this comment and will rephrase the discussion section to articulate clearly on factors that influence iodine status.

**Comment 2**: *Was the use of iodised salt investigated in the present study? What is the level of consumption among pregnant women? What is the availability and accessibility to iodised salt in the study areas?*

**Response**: We have noted these observations and, they will be part of the study's limitations.

**Comment 3**: *Authors indicated some strengths of the study; large sample size; but also identified limitations? One of the limitations was that they did not investigate use of iodised salt in the study area. This is a serious omission considering that iodised salt contributes to iodine intake and therefore UIC at least for a large part of the population. Additionally, there was no control group, which again raises concern over the design of this study.*

**Response**: Thank you so much for pointing this out. We will add more information that clarifies the limitation of our study design.

**Comment 4**: *Conclusion: It is a repeat of statement of results and not a conclusion. Authors should look at the objectives of the study and provide a concrete conclusion as to whether the objectives were attained and what are the implications to individual women and society in general.*

**Response**: We agree, and we will carefully rewrite the conclusion section based on the study objectives.

# Response

**Published:** 2022-08-23

**Authors:** Ray Masumo

**Affiliations:** Tanzania Food and Nutrition Centre, Tanzania

We appreciate the time and effort that you and the reviewer have dedicated to providing valuable feedback on our manuscript. Thank you very much.

 Here is a point-by-point response to the reviewers’ comments and concerns.

**Abstract**

**Comment 1:** *Use of the word 'insufficient' should be consistent throughout the article.*

**Response**: We agree with this comment and have incorporated your suggestion throughout the manuscript.

**Comment 2:** *Conclusion does not state explicitly the factors associated with insufficient/sufficient UIC*

**Response: **Thank you for pointing this out. We agree with this comment and have rewritten the conclusion as suggested.

**Introduction:**

**Comment 1:** *Provide explanation as to why highland regions have highest prevalence of IDD considering that Tanzania has been implementing salt iodisation programmes for many years?*

**Response:** Agree. We have revised the introduction section to clearly emphasize this point.

**Method:**

**Comment 1:** *Change 'Ante-natal Care Clinics' to 'Reproductive and Health Clinics' and use it consistently throughout the document.*

**Response:** We agree with this comment and have incorporated your suggestion throughout the manuscript.

**Comment 2:** *Explain adequately how sampling procedure was carried out.*

**Response**: We agree, and have rewritten the sampling procedures.

**Comment 3**: *Was the questionnaire pre-tested before translation?*

**Response: **Thank you very much for pointing this out. The questionnaire was pre-tested after translation.

**Comment 4:** *Interviews were done by health professional" - which cadre?*

**Response:** Interviews were done by a trained Nurse Midwife. We have incorporated this suggestion throughout the manuscript.

**Comment 5**: *Provide more information about the conditions for storage of the urinary samples in the field, transportation and storage at the laboratory before analysis and for how long were the samples stored before analysis. What distance from the field to the laboratory? This is important because iodine in urine is known to undergo changes after some time*.

**Response**: We agree with this comment and have added more information on the conditions of the urinary samples stored in the field, transportation, and storage at the laboratory before analysis.

**Comment 6**: *Was their any effort to ensure that women at various stages of pregnancy were well represented in the sample? It will also be useful to know of the sample size recruited, what was the stage of pregnancy for each participant? This information is missing*.

**Response**: Thank you very much for pointing this out. The inclusion criteria of this study clearly indicated that ‘ *To eliminate the effects of gestational age on thyroid hormone, participants beyond eight weeks of gestation were excluded*.

**Comment 7:** *Why Mbeya region? Provide justification.*

**Response:** Thank you for pointing this out and we have added the following statement in the manuscript for clarity. *This study is part of the project on improving maternal and adolescent nutrition in Mbeya supported by UNICEF Tanzania and the Ministry of Health- Tanzania*.

**Results** :

**Comment 1**: *Area of residence seemed to have an influence on iodine status of pregnant women, it was expected to see a good description of the study areas in this manuscript. The information is missing. Could the authors strive to provide this information?*

**Response**: Thank you for pointing this out and we have added a sub-section of the study site in the methods section.

**Comment 2**: *It will also be useful to know the stage of pregnancy (1st, 2nd, 3rd, trimester) of participants and if there were any association/interaction between area of residence, other factors and UIC.*

**Response**: You have raised an important point here. However, we believe that excluding participants of beyond eight weeks of gestation was more appropriate to avoid the effects of gestational age on thyroid hormone.

**Comment 3**: *Presentation of Tables should be improved; instead of brackets, authors could include columns for easy of reading*.

**Response**: We agree with this comment and have incorporated your suggestion.

**Comment 4**: *What was the consumption of iodised salt? This could be one of the contributing factors to high levels of UIC observed in some areas of the study. But also consumption of foods especially vegetables of the brassica group that are known to have compounds that interact with iodine in the gut.*

**Response**: Thank you for this suggestion. It would have been interesting to explore this aspect. However, in the case of our present study, the assessment of iodised salt and vegetables of the brassica group was out of scope and has been included in the limitation of the study.

**Comment 5**: *Clarification about consumption of processed meat*.

**Response**: Agree. Here we referred to processed meat as that which has been preserved by smoking, salting, curing, or addition of chemical preservatives. 

**Comment 6**: *Clearly stipulate factors that are associated with UIC in this section. Factors are not very clear.*

**Response**: We agree with this comment and have rewritten the result section to stipulate the factors associated with UIC.

**Discussion:**

**Comment 1**: *How were the identified factors influenced iodine status? These have not been adequately articulated in this manuscript*.

**Response**: We agree with this comment and have rewritten the discussion section to stipulate the identified factors that influence iodine status.

**Comment 2**: *Was the use of iodised salt investigated in the present study? What is the level of consumption among pregnant women? What is the availability and accessibility to iodised salt in the study areas?*

**Response**: You have raised an important point here. However, the use of iodised salt (availability and accessibility) was not investigated in this study, and we have included it as part of the study's limitations.

**Comment 3**: *Authors indicated some strengths of the study; large sample size; but also identified limitations? One of the limitations was that they did not investigate use of iodised salt in the study area. This is a serious omission considering that iodised salt contributes to iodine intake and therefore UIC at least for a large part of the population. Additionally, there was no control group, which again raises concern over the design of this study.*

**Response**: Thank you so much for pointing this out and, we highly appreciate the reviewers’ insightful and helpful comments on our manuscript. It would have been interesting to explore and we have included it in the study's limitations. We will add more information that clarifies the limitation of our study design.

**Comment 4**: *Conclusion: It is a repeat of the statement of results and not conclusion. Authors should look at the objectives of the study and provide a concrete conclusion as to whether the objectives were attained and what are the implications to individual women and society in general.*

**Response**: Thank you for pointing this out. We agree with this comment and have rewritten the conclusion as suggested.
